# Ovarian hyperstimulation syndrome with carotid artery dissection and cerebral infarction: a case report

**DOI:** 10.1186/s12905-023-02644-1

**Published:** 2023-09-19

**Authors:** Tianhua Fan, Liansheng Ma

**Affiliations:** Department of Neurology, The First Hospital of Shanxi Medical University, Taiyuan, Shanxi China

**Keywords:** Ovarian hyperstimulation syndrome, Carotid artery, Internal, Dissection, Brain infarction

## Abstract

**Background and purpose:**

Ovarian hyperstimulation syndrome (OHSS) is one of the most serious iatrogenic complications in assisted reproductive technology, which seem rarely associated with cerebrovascular diseases. We reported a patient diagnosed with OHSS combined with carotid artery dissection and massive cerebral infarction.

**Case presentation:**

We reported a unique case of a 31-year-old woman who experienced abdominal pain, blurred consciousness, and speech inability after 15-day continuous injection of human gonadotropin for infertility. Imaging examination showed hyperacute cerebral infarction in the left frontotemporal island parietal lobe and left internal carotid artery dissection. After therapeutic use of low-molecular-weight heparin calcium anticoagulation and other conventional cerebrovascular treatments, she eventually achieved a good prognosis.

**Conclusions:**

OHSS seemd rarely associated with cerebrovascular diseases, such as infarction and carotid artery dissection. Encountering abdominal symptoms combined with neurologic symptoms, a detailed history and a thorough examination are essential. It is necessary to comprehensively analyze the pathogenesis and formulate individualized therapy according to the specific conditions of patients.

## Introduction

Ovarian hyperstimulation syndrome (OHSS) refers to a series of clinical symptoms caused by the use of exogenous gonadotropin to overstimulate the ovary in assisted reproductive technology (ART), which is one of the most serious iatrogenic complications in ART. OHSS seems rarely associated with cerebrovascular diseases, such as infarction and carotid artery dissection, which may accompanied by high disability and mortality rate. We reported an infrequent case of a patient who was diagnosed with OHSS combined with carotid artery dissection and cerebral infarction, then analyzed its mechanism and countermeasures.

## Case presentation

A 31-year-old woman, 164 cm height and 80 kg weight, had been diagnosed with infertile and polycystic ovary syndrome (PCOS) for 2 years in the First Hospital of Shanxi Medical University. She had no other underlying diseases before and had not received any treatment after being diagnosed with infertility. 20 days before this admission was her first injection with ovulation stimulating needle and the protocol was 300−300−150IU of human gonadotropin for 3 cycles. She developed abdominal pain on the second day after the last injection. As the aggravating of abdominal pain, she was treated in Pingyao County Peoples’ Hospital (Pingyao, Shanxi) for symptomatic treatment (specific unknown).

**Fig. 1:**
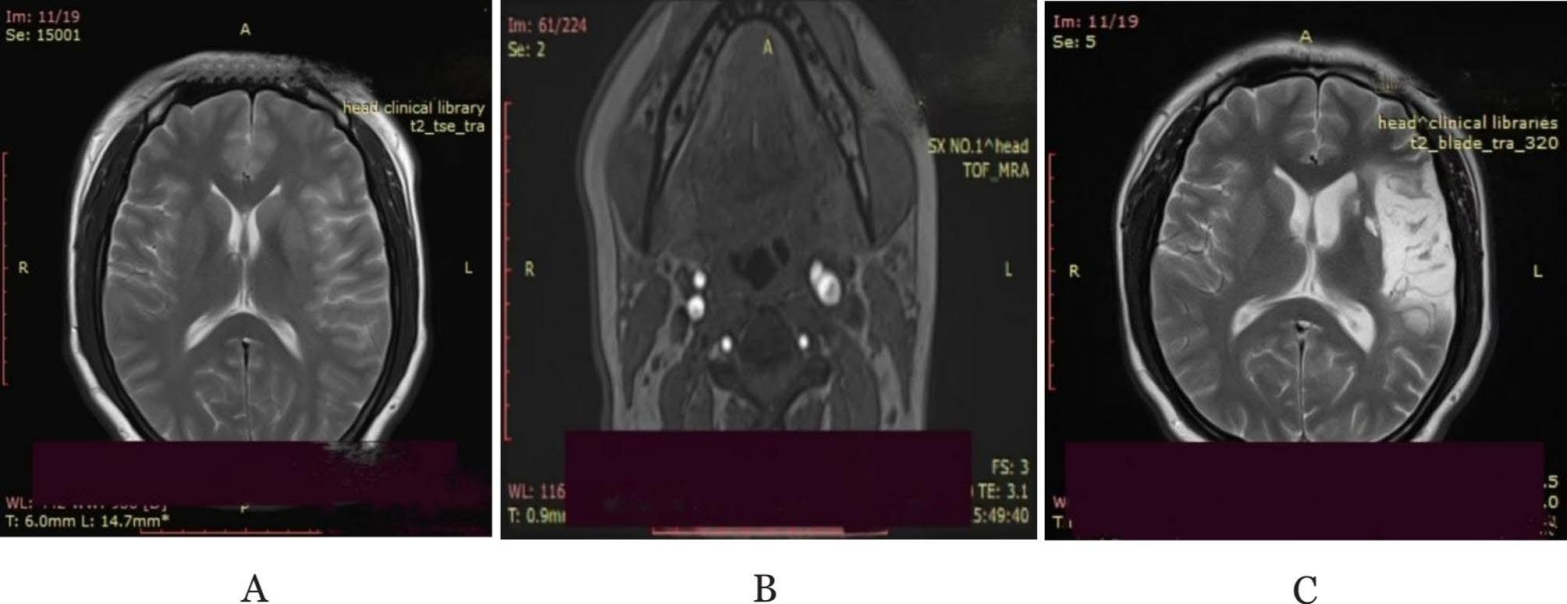
**A**: MRI showed hyperacute cerebral infarction in the left frontotemporal island parietal lobe; **B**: High-resolution magnetic resonance vessel wall imaging (HR-VWI) showed left ICA dissection; **C**: MRI showed no new infarctions than Figure A

**Fig. 2:**
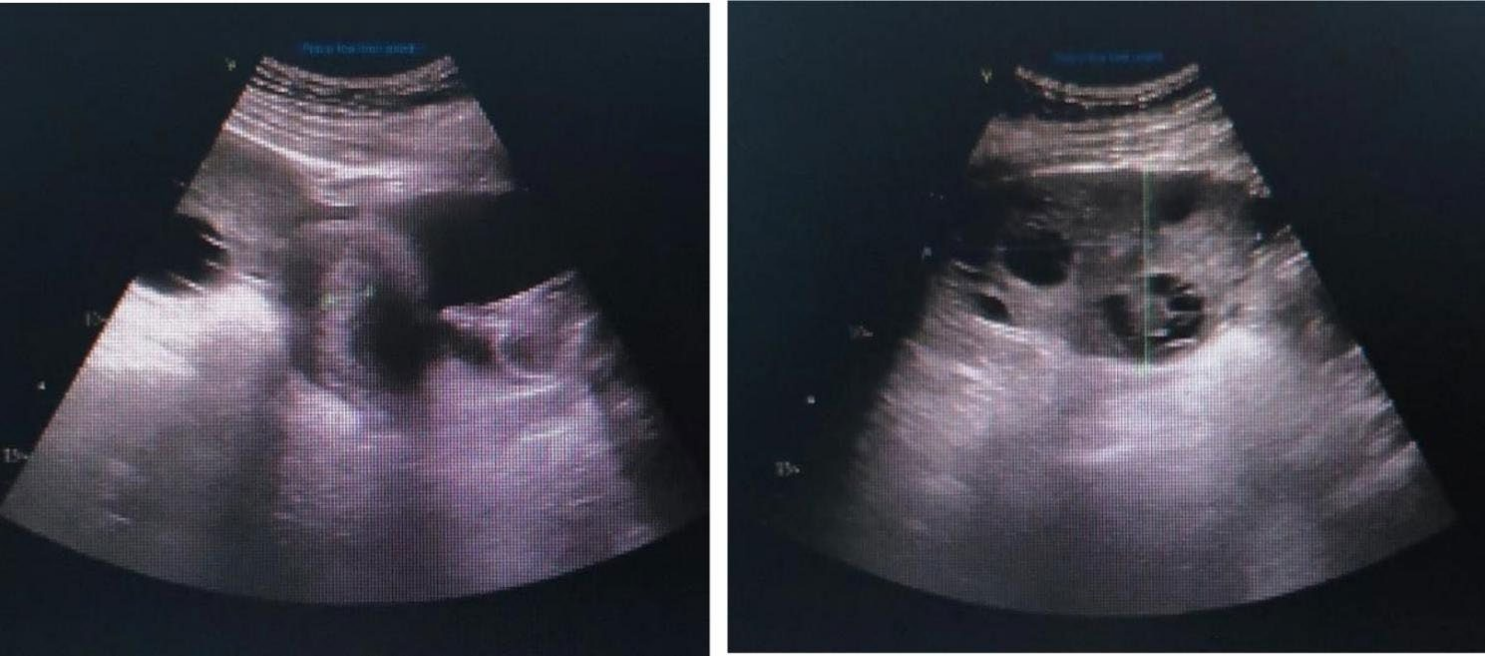
Bilateral ovarian volume increased

On the fourth day after the last injection, the patient developed distension, blurred consciousness, speech inability and paroxysmal binocular suspension, and was transferred to the emergency department of neurology of our hospital (The First Hospital of Shanxi Medical University, Taiyuan, Shanxi). General physical examination after admission revealed lower abdominal tenderness and mobile dullness. Positive neurological symptoms (Table 1) included drowsiness, mixed aphasia, right limb and facial hemiplegia,upper limb muscle strength worse than lower limb. Her National Institute of Health Stroke Scale Score (NIHSS) was 20. Laboratory investigations (Table 2) showed an elevated estradiol of 3000 pg/ml, β-hCG of 13.71 mIU/ml. Magnetic resonance imaging (MRI) showed hyperacute cerebral infarction in the left frontotemporal island parietal lobe (Fig. 1A); Magnetic resonance arteriography (MRA) revealed that the C2−7 segment of left internal carotid artery (ICA) and the M1 segment distal of left middle cerebral artery (MCA) were occluced; Gynecological color Doppler ultrasound showed increased bilateral ovarian volume and pelvic effusion, considering ovarian hyperstimulation (Fig. 2). Abdominal ultrasound showed fatty liver, intestinal effusion, and intestinal gas accumulation. Computed tomographic angiography (CTA) showed that the bulbar part of the left ICA was far away from the eye segment dissection, and the bifurcated upper trunk and distal part of the M1 segment of the left MCA was blocked. Computed tomographic perfusion (CTP) suggested ischemic changes in the left parietal lobe, temporal lobe, part of frontal lobe and occipital lobe. After comprehensive consideration of her condition, she was indwelling abdominal puncture drainage tubetreated and treated with nasal feeding tube 20 mg atorvastatin calcium tablets, subcutaneous injection of 0.5ml low-molecular-weight heparin calcium anticoagulation, intravenous infusion of 100ml butylphthalide and sodium chloride injection and 100ml 0.9% NaCI combined with 15ml edaravone and dexborneol concentrated solution for injection. She was transferred to our neurological intensive care unit for further treatment.

**Table 1:** Vital signs and positive signs of neurological examination

Time	Respiration	Heart rate	Blood pressure	Temperature	Positive signs
On admission	17 BPM	104 BPM	120/78mmHg	36.8℃	Drowsiness, Mixed aphasia, Right limb and facial hemiplegia,Bilateral Babinski sign (+)
Day1	14	84	108/64	36.4	Mixed aphasia, Stretch tongue to the right, Right limb and facial hemiplegia, Bilateral Babinski sign (+)
Day2	16	82	114/77	36.8	Incomplete mixed aphasia, Stretch tongue to the right, Right limb and facial hemiplegia, Right Babinski sign (+)
Day4	17	78	119/64	36.4	Incomplete mixed aphasia, Stretch tongue to the right, Right upper limb and facial hemiplegia, Right Babinski sign (+)
Day9	19	70	112/84	36.5	Speech is unfluent, can produce simple two-syllable vocabulary, Stretch tongue to the right, Right facial hemiplegia
Day28	18	75	122/88	36.4	Speech is unfluent, can produce simple alternative

**Fig. 3:**
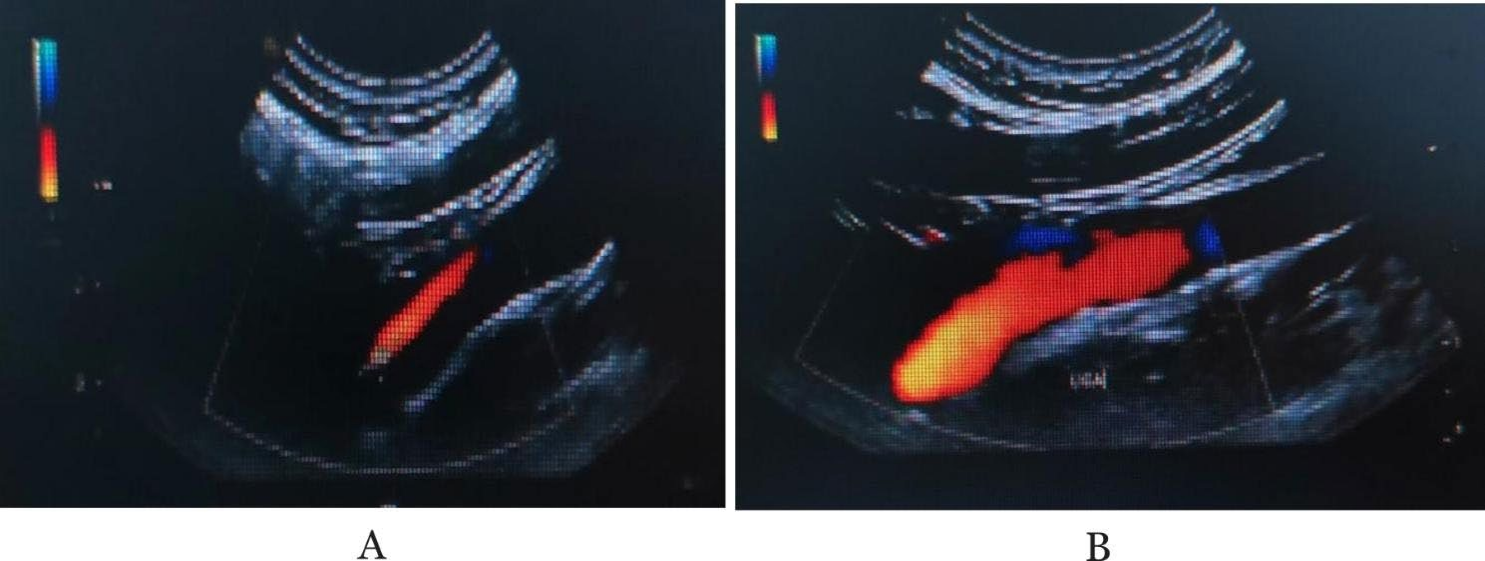
**A**: Acute thrombosis and dissection in the left ICA; **B**: Previous dissection has been completely repaired

On the first day after admission, her vital signs and consciousness status was better than that at admission. Carotid artery color doppler ultrasound showed acute thrombosis and dissection in the left ICA (intramural hematoma type) (Fig. 3A). Transcranial doppler (TCD) indicated left MCA stenosis (moderate to severe); TCD embolus monitoring showed positive microembolic signals in the left MCA, considering the existence of artery-artery embolism (dissecting intramural hematoma). On the basis of her original treatment, combined with subcutaneous injection of 5000 AXa low-molecular-weight heparin calcium anticoagulation and intravenous infusion of 50 ml albutein. Rehabilitation training was also added.

**Table 2:** Dynamic changes in laboratory investigations

Time	White blood cell (WBC)	Haematocrit	Platelet count	Albumin	Prothrombin time	D-dimer
1 day before admission	33.36*10^9/L	51.6%	430*10^9/L	29.1 g/L	15.2 s	-
On admission	21.8	43.8	365	27.2	16.7	1.64 mg/L
Day1	15.6	34.8	294	27.4	12.1	2.93
Day2	-	-	-	-	-	2.41
Day5	12.2	37.8	346	39.1	-	2.97
Day9	5.2	35.0	327	35.2	13.4	1.19

On the second day, she can partially understand our instruction, and can cooperate with the completion of eye opening and closing, lifting limbs. Magnetic resonance venography (MRV) showed no obvious abnormality. High-resolution magnetic resonance vessel wall imaging (HR-VWI) suggested left ICA dissection (Fig. 1B). The ultrasound results showed enlarged ovaries, peritoneal effusion and pelvic effusion. Combined with medical history, laboratory tests and examination, she was definitively diagnosed as OHSS complicated with left ICA dissection and massive cerebral infarction. Based on the above results, her abdominal drainage tubeshe was removed and she was treated with the previous treatment regimen and was transferred to the general ward of neurology.

On the fourth day, she could sit up on her own, and her right lower limb activity returned to normal. Cervical vascular ultrasound results were roughly the same as before. Her laboratory and general condition gradually recovered and was transferred to the rehabilitation department to continue rehabilitation treatment on the eighth.

After 17 days of rehabilitation training, she was discharged. Her physical activity was completely restored and she could communicate simply with others, but still unfluent. Re-examination of cervical vascular ultrasound showed the previous dissection has been completely repaired (Fig. 3B). After 3 months of discharge, she occasionally had difficulty in finding words, and the rest are normal. MRI suggested no new infarctions (Fig. 1C).

Written informed consent was obtained from the patient for the publication of this case report, including any potentially identifiable images or data included in this article.

## Discussion

OHSS often manifests as abdominal pain, abdominal distension, hypourocrinia, nausea, vomiting, electrolyte disturbance and weight gain. In severe cases, multiple organ failure, stroke and even death may occur. Studies have shown that ovulation-promoting drugs stimulate the ovary to secrete a large number of vascular endothelial growth factor (VEGF), inflammatory cytokines and vasoactive substances, which will cause vascular endothelial injury, fluid and protein extravasation from the blood vessels to the third space, resulting in blood concentration and blood volume reduction [1–4]. Extravasated liquid forms pleural effusion and ascites, which compress venous reflux and cause blood stasis. Both of these points can cause slow blood flow and increase the risk of thrombosis. In addition, high estrogen levels after ovulation induction treatment will lead to an imbalance between the coagulation system and the anticoagulant system, leaving the body in a hypercoagulable state and increasing the risk of thrombosis. OHSS may also increase vascular permeability by activating the renin-angiotensin system or reducing the production of VEGF inhibitors, leading to blood concentration thrombosis [5, 6].

The OHSS patient in this case were combined with massive cerebral infarction and ICA dissection. Previous studies have shown that vasoactive substances, inflammatory cytokines, significantly increased WBC may increase the risk of carotid artery dissection [7]. OHSS may cause vascular destruction and inflammation through the release of a large number of inflammatory cytokines and vasoactive substances, leading to vascular tear and carotid artery dissection. In addition, OHSS often causes changes in blood flow such as blood stasis, hypovolemic and hypercoagulability. Therefore, thrombosis caused by OHSS may also be one of the mechanisms of ICA dissection. However, we can not rule out the presence of vascular dysplasia in OHSS patients or the synergistic effect of the two mechanisms, which need to be further confirmed in subsequent studies .

Volume expansion should be placed in the primary and key position in the treatment of OHSS, which may avoid multiple organ failure caused by low perfusion. The selection of anticoagulants and antiplatelet agents should be individualized according to the possible pathogenesis of patients. Some studies have shown that the combination of heparin and antiplatelet drugs in the initial stage of OHSS may achieve better results and leaving no adverse consequences. It is uncertain whether intravenous thrombolysis and mechanical thrombectomy are feasible. Previous studies have listed intravenous thrombolysis as one of the contraindications during pregnancy. However, some cases reported that patients achieved good results after intravenous thrombolysis and mechanical thrombectomy [8]. Therefore, appropriate therapy for OHSS depends on the condition of the patients. In addition, the addition of statins in the early stage of OHSS with carotid artery dissection and stroke will play an endothelial protective role and avoid the progression of arterial dissection and stroke to a certain extent. In addition to the above treatment, symptomatic treatments are also critical in OHSS treatment.

In conclusion, OHSS seemed rarely associated with cerebrovascular diseases, such as infarction and carotid artery dissection. Encountering abdominal symptoms combined with neurologic symptoms, a detailed history and a thorough examination are essential. Volume expansion is a key. In addition, it is necessary to comprehensively analyze the pathogenesis to formulate individualized conventional cerebrovascular treatments.

## Data Availability

The datasets used and/or analysed during the current study available from the corresponding author on reasonable request.

## Abbreviations

OHSS: Ovarian hyperstimulation syndrome
ART: Assisted reproductive technology
PCOS: Polycystic ovary syndrome
NIHSS: National Institute of Health Stroke Scale Score
MRI: Magnetic resonance imaging
MRA: Magnetic resonance arteriography
ICA: Internal carotid artery
MCA: Middle cerebral artery
CTA: Computed tomographic angiography
CTP: Computed tomographic perfusion
TCD: Transcranial doppler
MRV: Magnetic resonance venography
HR-VWI: High-resolution magnetic resonance vessel wall imaging
VEGF: Vascular endothelial growth factor
